# Usability testing of two co-designed discharge communication tools for use in pediatric emergency departments: findings from the EDUCATE study

**DOI:** 10.1186/s12887-026-06916-1

**Published:** 2026-04-23

**Authors:** Mari Somerville, Lori Wozney, Allyson J Gallant, Amy Plint, Kristina Vogel, Rebecca Mackay, Stephanie Rowe, Christine Cassidy, Alannah Delahunty-Pike, Janet A Curran

**Affiliations:** 1 IWK Health, Halifax Nova Scotia, 5980 University Avenue #5850, Halifax , Nova Scotia B3K 6R8 Canada; 2 Nova Scotia Health, 90 Lovett Lake Court, Halifax, NS B3S 0H6 Canada; 3 Faculty of Health, Dalhousie University, Room 316-5968 College Street, PO BOX 15000, Halifax, NS B3H 4R2 Canada; 4 CHEO Research Institute, 401 Smyth Road- Research Building 2, 2nd floor, Room 2119, K1H 8L1 Ottawa, Ontario (ON) Canada; 5 School of Nursing, Dalhousie University, Halifax, NS Canada

**Keywords:** Usability test, Digital health, Emergency department, Pediatrics, Asthma, Concussion

## Abstract

**Background:**

Discharge communication is a key component of high-quality care. This practice is important in the pediatric emergency department (PED), where patients and families often leave without fully understanding important information shared during their visit. Co-designed discharge communication interventions may enhance information exchange in PEDs. The aim of this study was to assess the usability of two discharge communication tools, co-designed with youth, parents, and PED clinicians, for asthma or concussion presentations.

**Methods:**

This was a mixed methods study with a concurrent validating quantitative triangulation design. Youth, parents, and clinicians recruited from two Canadian PEDs completed usability tests for either an asthma or concussion co-designed discharge communication intervention. Usability Testing Round 1 was remote, where participants provided quantitative and qualitative feedback on tool functionality. Feedback from Round 1 informed the revised tools for Round 2 testing. Remote Usability Testing Round 2 was self-administered by a new group of participants who provided feedback on user experience and satisfaction with the tool by completing 5-point Likert scale questions and open-text fields. Round 1 data were analyzed using the severity of usability problems (SUP) scoring. Quantitative data from Rounds 1 and 2 were analyzed descriptively. Deductive content analysis was applied to qualitative data from Rounds 1 and 2. One reviewer coded participant feedback and a second reviewer verified categories.

**Results:**

Round 1 (*n* = 14) revealed primarily cosmetic and minor usability issues for each tool. Revised tools were used in Usability Testing Round 2 (*n* = 40), which identified additional, minor revisions to each tool. A key suggestion was offering the tools in multiple languages and formats to ensure they met the diverse needs of patients and families presenting to the PED. Site 1 participants consistently scored the tools more helpful or useful compared to Site 2.

**Conclusions:**

Through two rounds of comprehensive usability testing, we refined each tool to meet the needs of youth, parents and PED clinicians. Enhanced recruitment strategies are needed to reach youth and diverse populations to ensure future tools meet a range of information needs during PED discharge.

**Supplementary Information:**

The online version contains supplementary material available at 10.1186/s12887-026-06916-1.

## Background

Discharge communication should provide a summary of patients’ healthcare interactions and important follow-up instructions [1], and is a key aspect of patient-centred care [2]. However, there is a lack of guidance for clinicians to provide high quality, effective discharge communication [1, 3]. Discharge communication can be impacted by a range of systemic, interpersonal and patient specific factors [4, 5]. This is particularly true in the pediatric emergency department (PED), which can be a noisy and chaotic environment [6]. Poor discharge communication can result in individuals leaving the PED without fully understanding their discharge care instructions [6, 7]. Without proper discharge communication, patients can experience poor health outcomes, uncertainty about their condition, and make unnecessary return visits to the PED [8, 9].

Asthma and concussion are common PED presentations [10]. These conditions are also associated with a high number of return PED or adult ED visits due to a poor understanding of discharge instructions [11, 12]. Discharge communication is critical in a pediatric population as parents (i.e., parents, caregivers or legal guardians) are often responsible for continuing their child’s care at home. However, an inadequate understanding of PED discharge communication can affect their confidence to manage their child’s symptoms and follow-up care [13]. Due to the high number of asthma and concussion PED presentations each year, it is crucial to ensure proper discharge communication targets the needs of these three population groups.

Discharge instructions have traditionally been delivered through verbal and/or written formats [14]. However, patients and clinicians have identified a preference for digital discharge communication practices [15, 16]. A survey of youth living with asthma reported high levels of satisfaction with a digital self-management tool [17], while another study identified a digital concussion tool was well received by youth [18]. Electronic discharge communication tools implemented in PEDs have been successfully integrated with usual care for a variety of illness presentations, including asthma and head injury [19]. While this evidence presents a strong base for designing future discharge communication interventions, few included studies examined clinician or patient experiences [14, 19], which highlights a gap in user-centred design. Further, a review of 170 digital health interventions identified only 30% included usability testing in their development [20], despite usability testing being recognized as a crucial aspect of intervention design to ensure real world effectiveness [21, 22].

To address these gaps in the PED and health intervention literature, we conducted a co-design study to develop two *e*mergency department *d*ischarge comm*u*nication str*ate*gies (EDUCATE) with pediatric patients, parents, and PED clinicians [23]. The objective of the current study was to assess the usability of the two co-designed EDUCATE tool prototypes, designed for children and youth presenting to PEDs with asthma or concussion.

## Methods

### Study design & setting

This mixed methods study used a concurrent validating quantitative triangulation design [24]. This approach involves collecting both quantitative and qualitative data simultaneously and analyzing separately. The qualitative findings are used to validate the quantitative data and do not require an intensive qualitative analysis step [25]. This study took place remotely, with participants recruited from two urban PED sites in Canada. Ethics approval was obtained from each institution’s research ethics board prior to data collection (Site 1 Reference: 1024004; Site 2: REB: 21/62X). This study presents findings from the usability testing stage of the broader EDUCATE study, where two electronic discharge communication tools were co-designed by youth, parents, and clinicians [23, 26, 27]. Findings were reported using the good reporting of a mixed methods study (GRAMMS) guidelines [28].

### Tool development

As part of the broader EDUCATE study, two discharge communication tools were developed through a co-design process [26]. A co-design team was formed for each PED illness presentation (i.e., asthma and concussion). Each team included parents and youth with lived experience visiting the PED for asthma or concussion, and PED clinicians who provide care for these conditions.

Across eight intervention design and refinement meetings, the asthma team co-designed an electronic tool to help individuals decide when to visit the PED during an asthma attack. The concussion co-design team developed a tool to help individuals monitor post-concussion symptoms at home following PED discharge [26]. This team suggested the concussion tool be available in electronic and paper formats to accommodate the reduced screen time requirements commonly associated with concussion management.

Following intervention co-design meetings, user experience design experts created a tool prototype for each team that aligned with each co-design team’s identified aim. The asthma tool used a traffic light system to gauge severity of asthma symptoms and when to attend PED (red), schedule an appointment with family doctor (yellow) or continue to avoid triggers and monitor asthma symptoms at home (green). The concussion tool had an online, interactive concussion symptom tracker to allow collection of activities, symptom intensity and comments. Images of the tool prototypes can be found in supplementary file 1. Version 1 of these tools were subjected to Usability Testing Round 1. Following initial usability testing findings each tool was refined, and Version 2 was tested in Usability Testing Round 2.

### Participants

Nielsen and Landauer (1993) found that 85% of usability issues could be identified with as few as five participants [29]. Formative usability tests are also better suited to smaller numbers, as testing involves an iterative process of usability testing, refinement and re-testing, with a prototype tool [30]. Therefore, we proposed a sample of 2–3 participants per target population group (i.e., youth, parents, clinicians) for each condition (i.e., asthma, concussion), from each PED site, for a total of 16–24 participants per round of usability testing.

### Recruitment & screening

To achieve a diverse sample of 16–24 participants per round of testing, youth and parent participants were recruited through social media outlets, in-person recruitment posters, hospital volunteers and announcements from PED staff. Local sports organizations also shared recruitment details with their membership. Clinicians were recruited through centre-wide emails and in-person announcements. Snowball sampling was used with clinical research team members sharing study details directly with colleagues.

In Usability Testing Round 1, interested individuals completed an on-line screening survey administered through the Research Electronic Data Capture (REDCap) platform [31]. The screening survey included questions about participant’s role, experience working in or visiting a PED, and PED illness presentation (i.e., asthma or concussion). Individuals who met inclusion criteria received a consent form and an invitation to complete a usability test with the lead researcher on a day and time convenient for them. Study personnel monitored the screening surveys and adapted their recruitment approach to ensure representation from each target group (i.e., youth, parents, clinicians) and for each condition (i.e., asthma, concussion). Recruitment stopped once the target sample size was met (i.e., 2–3 participants per target group). Individuals who completed the screening survey after capacity was reached were given the opportunity to participate in Usability Testing Round 2.

Using the same recruitment approach in Round 2, interested participants completed an online screening survey through REDCap to determine eligibility. Eligible participants then provided written, informed consent through REDCap prior to completing the usability test. Study personnel monitored survey responses and used branching logic in REDCap to close the survey for individual target groups once capacity was reached (i.e., 2–3 participants per target group).

### Data collection: Usability Testing Round 1 & 2

A detailed description of the usability testing process and measures used were previously described [27] and are briefly outlined below.

#### Round 1

The lead author (MS) facilitated all virtual, synchronous usability tests in Round 1, which were held and recorded using Zoom Video Communications software (Zoom Video Communications Inc., San Jose, USA). The usability approach was modified depending on the participant group (i.e., youth, parent, clinician) and condition (i.e., asthma, concussion) [27]. Participants consented to having the session video recorded but had the option to keep their camera off and use an alias to protect their identity.

Round 1 included Site 1 participants and took place between December 2021 and July 2022 using Version 1 prototypes of each EDUCATE tool. Following a brief overview of the study procedures, expectations and rights, participants completed a pre-test survey to collect demographics. Next, the facilitator explained and provided an example of the ‘think aloud’ process to participants, a method where individuals describe their thoughts as they complete tasks [32]. Youth participants watched an additional video of a youth completing a think aloud process to enhance their comfortability with the process. The facilitator then shared a link to Version 1 of the EDUCATE tool being tested and asked participants to share their screen.

Participants were then instructed to open the tool and asked to describe their first impressions by selecting from a list of descriptive words. Several quantitative and qualitative measures were then used to collect data. This included having participants think aloud while completing a series of questions and two scenarios to test the functionality of the tool (e.g., using tool to decide to attend PED with asthma attack, or tracking concussion symptoms, or treating asthma/concussion). At the end of the session, participants completed a post-test survey off-camera with the facilitator present. The post-test survey included a series of questions (e.g., the tool held my attention, I like the illustrations, it is easy to understand) adapted from Gibson et al. (1991) [33]. Each item was scored on a 5-point Likert scale (1 = strongly disagree; 5 = strongly agree) with a textbox to gather additional qualitative data on usability insights. Participants were offered a $30 gift card for their time.

#### Round 2

In line with formative usability testing, Round 2 involved a less intensive approach. Data were collected asynchronously and remotely through REDCap survey responses, as opposed to individual virtual meetings. Usability Testing Round 2 included participants from PED Sites 1 and 2 and took place between November 2022 and March 2023. Through the online surveys, participants answered demographic questions and reviewed embedded screenshots and video clips of the tool. Based on these images and clips, participants answered five (concussion surveys) or seven items (asthma surveys) about the tool’s usability, helpfulness and purpose on 5-point Likert scales (1 = strongly disagree; 5 = strongly agree). Participants could provide additional feedback through open-text comment boxes in each survey. Participants received a $30 gift card for completing the survey.

### Data analysis: Usability Testing Round 1 & 2

Following a concurrent validating quantitative triangulation design [25], quantitative and qualitative data were analyzed separately but presented together to provide a comprehensive overview of the usability of each tool. Round 1 data were analyzed first, followed by Round 2 data collection and analysis.

Quantitative survey data and participant demographics were descriptively analyzed and presented as frequencies and percentages. Data were presented in tables or figures where appropriate to show differences between condition types (Round 1 & 2) and sites (Round 2). Common descriptive words identified by participants in Usability Testing Round 1 were used to generate a Word Cloud for each tool (supplementary file 2).

Round 1 qualitative data were transcribed verbatim and Nielson’s severity of usability problems (SUP) scoring criteria [34] informed the content analysis step. One researcher (MS) coded each participant comment by assigning usability errors a SUP score between zero (e.g., no issue) to four (e.g., catastrophic issue) [34]. 50% of the coded usability errors and SUP scores were then verified by a second coder (AG, JAC, LW). Any conflicts between SUP scores were resolved through discussion between coders. A final list of SUP scores and exemplar quotes were presented in tables. Additional participant quotes were deductively coded and analyzed using the three categories: (i) overall satisfaction; (ii) decision supports and content; and (iii) suggestions for improvement. A narrative description was presented for each tool with additional quotes included as supplementary material. This feedback was used to inform tool revisions prior to Round 2 testing.

Following a content analysis approach, Round 2 survey responses were deductively coded and analyzed using the same three categories as Round 1. One researcher (MS) transcribed the open-text responses from each survey into tables. Trends in feedback were coded by MS and verified by a second coder. Theme descriptions were presented narratively with more detailed quotes included as supplementary material.

## Results

Fifty-four participants took part across both rounds of Usability Testing; 14 completed Usability Testing Round 1 (asthma: *n* = 7; concussion: *n* = 7), with 40 additional participants included in Round 2 (asthma: *n* = 21; concussion: *n* = 19).

### Round 1 usability tests

Participant demographic details from both clinical groups (i.e., asthma and concussion) are found in Table 1. Participants’ first impressions of the tools were positive, with all asthma participants (*n* = 7; 100%) describing it as useful, 57% described it as helpful and 57% described it as valuable. Concussion participants (*n* = 7) provided almost identical scores, with 100% of participants describing their tool as useful, 57% described it as helpful, 57% said it was clear and 57% described it as simplistic (See Supplementary File 2).

**Table 1:** Demographic data for Usability Testing Round 1 for asthma and concussion

Characteristic	Asthma Participants (*n* = 7) *N*(%)	Concussion Participants (*n* = 7) *N*(%)
Usability test in past 6 months? Yes No	0 (0) 7 (100)	1 (14.3) 6 (85.7)
Ethnicity Caucasian Asian Black or African Nova Scotian First Nation Prefer to Self-describe	5 (71.4) 0 (0.0) 0 (0.0) 0 (0.0) 2 (28.6)	6 (85.7) 1 (14.3) 0 (0.0) 0 (0.0) 0 (0.0)
Highest Education Level Attained Elementary or middle school Some high school High school diploma College diploma University degree Postgraduate degree (Masters, PhD, MD) Other	0 (0.0) 0 (0.0) 1 (14.2) 0 (0.0) 3 (42.9) 3 (42.9) 0 (0.0)	0 (0.0) 2 (28.6) 0 (0.0) 0 (0.0) 4 (57.1) 1 (14.3) 0 (0.0)
Typical Activities on the Computer* (Check all that apply) Work-related activities Social media Creative School-related activities Leisure activities	7 (100.0) 5 (71.4) 0 (0.0) 3 (42.9) 5 (71.4)	6 (85.7) 6 (85.7) 1 (14.2) 5 (71.4) 7 (100.0)
Access Websites for Health-related Activities? Yes No	6 (85.7) 1 (14.3)	6 (85.7) 1 (14.3)
Confidence from 1–10 in using health-related websites/apps Mean (SD)	8 (2.0)	8 (1.6)
How do you access the internet? Mostly on my phone Mostly on a computer/laptop/tablet About equally on a phone/computer/laptop/tablet Neither phone or computer/laptop/tablet	3 (42.9) 1 (14.2) 3 (42.9) 0 (0.0)	4 (57.1) 1 (14.3) 2 (28.6) 0 (0.0)
Role Youth Parent Nurse Physician	2 (28.6) 2 (28.6) 2 (28.6) 1 (14.3)	2 (28.6) 1 (14.3) 3 (42.8) 1 (14.3)
**Clinicians**	(*n* ** = 3)**	(*n* ** = 4)**
Time in Position < 5 years 5–10 years > 10 years	0 (0.0) 1 (33.3) 2 (66.6)	2 (50.0) 0 (0.0) 2 (50.0)
Average Number of PED Shifts Once or more per week Once or more per month Once every few months Never	1 (33.3) 2 (66.6) 0 (0.0) 0 (0.0)	3 (75.0) 1 (25.0) 0 (0.0) 0 (0.0)
Frequency of Asthma/Concussion Presentation More than once per week Weekly More than once per month Monthly Once every few months Never	1 (33.3) 0 (0.0) 2 (66.6) 0 (0.0) 0 (0.0) 0 (0.0)	3 (75.0) 1 (25.0) 0 (0.0) 0 (0.0) 0 (0.0) 0 (0.0)
Confidence in providing discharge communication (1–10) Mean (SD)	9 (1.5)	8.5 (1.3)
**Parents and Youth**	(*n* ** = 4)**	(*n* ** = 3)**
Last Visit to PED for Asthma/Concussion Less than 1 week ago Less than 1 month ago 1–2 months ago More than 2 months ago	0 (0.0) 0 (0.0) 3 (75.0) 1 (25.0)	1 (33.3) 0 (0.0) 0 (0.0) 2 (66.6)
First visit to the PED for asthma/concussion? Yes No	1 (25.0) 3 (75.0)	2 (66.6) 1 (33.33)
How long have you/your child been living with asthma/concussion? A few months About 1 year Between 1–2 years Between 2–5 years More than 5 years	0 (0.0) 1 (25.0) 0 (0.0) 1 (25.0) 2 (50.0)	1 (33.3) 0 (0.0) 1 (33.3) 1 (33.3) 0 (0.0)

*Participants eligible to select one or more responses; therefore, response totals may exceed N

#### Asthma tool

Two youth, two parents, two PED nurses, and one physician tested Version 1 of the asthma tool. Testing sessions took between 36 and 76 min to complete (mean: 51.5 min; Standard Deviation [sd]: +/- 13.1). A usability issue was coded 105 times across all participants, with issues receiving SUP scores between 1 (cosmetic; e.g., font size, colours used) and 4 (catastrophic; e.g., key information missing from page). Most usability problems were classified as cosmetic (SUP score 1; *n* = 53; 50.5%) or minor usability problems (SUP score 2; *n* = 40; 33.1%). Most usability problems were identified within the first five minutes of clicking around the tool and were found on the main landing page (Table 2).

**Table 2:** Usability Testing Round 1 SUP scores and corresponding suggestion for the asthma tool

Task/Question	Frequency of SUP Scores SUP# (*n*; %)	Example of suggested changes with SUP score
Part 1 – First impressions and self-directed use of tool		
Main page (*n* = 41)	SUP 0 (1; 2.4%) SUP 1 (21; 51.2%) SUP 2 (14; 34.1%) SUP 3 (5; 12.2%) SUP 4 (0; 0%)	1 – In red zone, it should be bolded where it says ‘any of these symptoms’ 2 – could add a breathing technique video (something that shows timing of breaths to breathe along to) 3 – confusion about why some buttons have checkmarks and others don’t
Education Page: Symptoms (*n* = 26)	SUP 0 (0; 0%) SUP 1 (11; 42.3%) SUP 2 (11; 42.3%) SUP 3 (4; 15.4%) SUP 4 (0; 0%)	1 – wheezing sound clip should have a stop option 2 – first part of wheezing clip doesn’t sound right 3 – symptoms buttons not working
Education Page: Triggers (*n* = 8)	SUP 0 (2; 25%) SUP 1 (4; 50%) SUP 2 (2; 25%) SUP 3 (0; 0%) SUP 4 (0; 0%)	0 – nothing, I really like this page 1 – add a cross through cigarettes icon 2 – could make it more interactive by getting the lungs to move
Education Page: Follow-up (*n* = 11)	SUP 0 (0; 0%) SUP 1 (9; 81.8%)) SUP 2 (2;18.2%) SUP 3 (0; 0%) SUP 4 (0; 0%)	1 – text could be slightly bigger 2 – care action plan page is useful and important but I’m not drawn to the page
Part 2 – Task-based scenarios		
Task 1 (*n* = 12)	SUP 0 (1; 8.3%) SUP 1 (2; 16.7%) SUP 2 (6; 50%) SUP 3 (3; 25%) SUP 4 (0; 0%)	0 – no changes needed 1 – need to consider different languages 2 – some uncertainty about why some checkmarks are clicked 3 – should add information about medication and how to properly give the inhaler
Task 2 (*n* = 11)	SUP 0 (0; 0%) SUP 1 (6; 54.5%) SUP 2 (5; 45.5%) SUP 3 (0; 0%) SUP 4 (0; 0%)	1 – different wording for ‘tracheal tug’ 2 – some navigation issues when going from main page directly to tracheal tug

*SUP* Severity of Usability Problems

supplementary file 3 provides participants’ post-test survey results. Participants were asked to use the tool to decide whether to go to the PED. This task revealed several usability problems, which all received SUP scores between 1 (cosmetic; e.g., content layout) and 3 (major problem; e.g., unable to click buttons in tool). One participant also reported that they would not follow the advice given in the tool and would go to their doctor immediately, rather than waiting a few days as suggested in the tool (SUP score of 3). Of the seven participants, five correctly selected the yellow zone for their scenario-based task. Four of these participants (*n* = 4/5; 80%) correctly decided to wait to go to the PED, while one person decided to go to the PED despite the directions provided in the tool. The remaining two participants did not complete the task and instead navigated to other areas of the tool.

Qualitative data were categorized into one of three groups: (i) overall satisfaction; (ii) decision supports and content; and (iii) suggestions for improvement. An overview of this feedback is outlined below, with additional detail and participant quotes included as supplementary file 4.

- *Overall Satisfaction*. Participants generally found the asthma tool to be useful and important. Parents and youth especially described their overall satisfaction with the tool. Some aspects of the asthma tool were clearly confusing to participants and were therefore coded as a frequent usability issue. For example, several participants commented about the fact that some of the buttons were ‘checked’ on the asthma symptom checklist while others were not. Participants were unsure if there was any meaning behind this or whether it was meant to show that the buttons could be interactive.
- *Decision Supports and Content*. The main page of the tool, which included a traffic light system of green, yellow and red zones for deciding when to go to the PED during an asthma attack, had the most usability errors (SUP scores of 1-2). The ‘Symptoms’ page had several usability issues (n=26; 24.8%), again with most being cosmetic or minor concerns (n=22;84.6%). Participants liked the videos and/or sound clips of symptoms, yet several people wanted additional symptom sound clips or details.
- *Suggestions for Improvement*. Post-test survey open-ended responses indicated participants liked the tool overall but had suggestions for improvements. Several clinicians reported offering a tool in additional languages and formats to be more accessible to different PED populations. Another suggestion was to include an education page with information about asthma and common medications used in treatment. These changes were made during the refinement stage and education pages were included in Version 2 of the tool, which was assessed in Usability Testing Round 2.

### Concussion tool

Two youth, one parent, three PED nurses, and one physician completed testing with Version 1 of the concussion tool. Usability testing sessions took between 33 and 61 min (mean; 44.0 min; SD: +/- 10.8) to complete. A usability issue was coded 89 times across all participants, with SUP scores of 1 (cosmetic) to 3 (major issue; unable to click buttons). Most usability problems were identified on the ‘symptom tracker’ (*n* = 29; 32.6%) and ‘recovery plan’ pages (*n* = 23; 25.8%) and were considered cosmetic or minor issues (SUP = 1–2). No usability issues were coded as catastrophic (SUP = 4; Table 3).

**Table 3:** Usability Testing Round 1 SUP scores and corresponding suggestions for the concussion tool

Task/Question	Frequency of SUP Scores SUP # (*n*)	Example of suggested changes
Part 1 – First Impressions		
Main Page (*n* = 12)	SUP 0 (2; 16.7%) SUP 1 (7; 53.3%) SUP 2 (3; 25.0%) SUP 3 (0; 0%) SUP 4 (0; 0%)	0 – I like how it says developed by [Site 1 and academic institution], but I would personally make a new line for each one 1 – suggest to change ‘sudden shaking of the head’ wording to something that makes more sense to parents 2 – struggling to scroll to bottom of page
Returning to Emergency Page (*n* = 20)	SUP 0 (5; 25.0%) SUP 1 (12; 60.0% ) SUP 2 (3; 15.0%) SUP 3 (0; 0%) SUP 4 (0; 0%)	0 – nothing to say on the ‘red flag’ symptoms; I like it overall and it is in laymen terms 1 – can clarify some symptoms (e.g., Under ‘very confused’ can say ‘can’t talk, hard to remember name/facts’) 2 – Vomiting repeatedly is not going to cut it; We usually say ‘vomiting 5–6 times’ because otherwise we get people coming in who have vomited twice
Recovery Plan Page (*n* = 23)	UP 0 (4; 17.4%) SUP 1 (17; 79.3%) SUP 2 (2; 8.6%) SUP 3 (0; 0%) SUP 4 (0; 0%)	0 – really like the recovery plan as it is in line with what I was told in the ED 1 – change wording where it says ‘you need a doctors note’ as people might think they need to come back to emerg for that 2 – could be helpful if you could have a way to click the symptoms and it told you what to do
Symptom Tracker (*n* = 24)	SUP 0 (7; 29.2%) SUP 1 (15; 62.5%) SUP 2 (2; 8.3%) SUP 3 (0; 0%) SUP 4 (0; 0%)	0 – oh record your symptoms, this is really cool! I really like this 1 – add option for being on your phone or electronics under ‘activity’ 2 – needs some more instruction about why to use the symptom tracker, otherwise wouldn’t use it
**Part 2: Scenario based questions**		
Task 1 (*n* = 17)	SUP 0 (4; 23.5%) SUP 1 (7; 41.1%) SUP 2 (6; 35.3%) SUP 3 (0; 0%) SUP 4 (0; 0%)	0 – I think it’s great because it’s so self-explanatory 1 – put recovery plan and symptom tracker in larger letters on main page 2 – Buttons not working when trying to complete task
Task 2 (*n* = 19)	SUP 0 (4; 21.1%) SUP 1 (12; 63.2%) SUP 2 (3; 15.8%) SUP 3 (0; 0%) SUP 4 (0; 0%)	0 – really liked that it’s very clearly laid out and goes through all the symptoms 1 – could change the colours to be more severe/red at the top and fade to less serious 2 – rather than directing people to go straight to hospital under ‘returning to ED’, could direct people to exercises to try before going to emerg

*SUP* Severity of Usability Problems

A usability scenario task asked participants to use the symptom tracker to record their symptoms and decide whether to attend the PED. Participants generally found the tool helpful for completing this task, with some suggestions coded as cosmetic or minor usability issues. Additional qualitative data, coded into the three categories described previously, are outlined below with additional quotes in Supplementary File 4.

- *Overall Satisfaction*. Participants described the tool favorably and liked the symptom tracker and recovery guide sections of the tool. A few participants commented on the value of the tool in alleviating some of the challenges related to PED discharge communication.
- *Decision Supports and Content*. Participants also appreciated that the tool features were specific to the needs of those in concussion recovery. Some participants had concerns about wording on the ‘symptom tracker’ page. One parent was unclear of the purpose of the symptom tracker and may not use it at home unless instructed by a clinician.
- *Suggestions for Improvement*. The key suggestion for improvement from participants was clarifying details of concussion symptoms.

### Round 2 usability tests

Forty participants completed Usability Testing Round 2 across both participating sites (asthma: *n* = 21 [52.5%]; concussion: *n* = 19 [47.5%]). Overall, participants tended to be Caucasian (*n* = 30/40; 95%), identified as female (*n* = 29/40; 72.5%) and spoke English as their first language (*n* = 38/40; 95%). Participant demographic details for both clinical groups (i.e., asthma and concussion) and sites are found in Table 4.

**Table 4:** Demographic details of Usability Testing Round 2 participants

Characteristic	Asthma Participants		Concussion Participants	
	Site 1 PED *N*(%)	Site 2 PED *N*(%)	Site 1 PED *N*(%)	Site 2 PED *N*(%)
	(*n* = 12)	(*n* = 9)	(*n* = 10)	(*n* = 9)
Age < 12 years 12–19 years 20–29 years 30–39 years 40–49 years > 50 years	0 (0.0) 1 (8.3) 3 (25.0) 6 (50.0) 1 (8.3) 1 (8.3)	0 (0.0) 2 (22.2) 1 (11.1) 3 (33.3) 1 (11.1) 2 (22.2)	0 (0.0) 0 (0.0) 6 (60.0) 2 (20.0) 1 (10.0) 1 (10.0)	0 (0.0) 1 (11.1) 0 (0.0) 5 (55.5) 2 (22.2) 1 (11.1)
Gender Male Female Prefer to self-describe	2 (16.7) 10 (83.3) 0 (0.0)	2 (22.2) 7 (77.8) 0 (0.0)	2 (20.0) 8 (80.0) 0 (0.0)	5 (55.6) 4 (44.4) 0 (0.0)
Race/Ethnicity Caucasian Asian Black or African Canadian First Nation Prefer to Self-describe	10 (83.3) 2 (16.7) 0 (0.0) 0 (0.0) 0 (0.0)	4 (44.4) 0 (0.0) 4 (44.4) 0 (0.0) 1 (11.1)	10 (100.0) 0 (0.0) 0 (0.0) 0 (0.0) 0 (0.0)	6 (66.7) 0 (0.0) 1 (11.1) 0 (0.0) 2 (22.2)
English as First Language Yes No	12 (100.0) 0 (0.0)	8 (88.9) 1 (11.1)	10 (100.0) 0 (0.0)	8 (88.9) 1 (11.1)
Typical Activities on the Computer (Check all that apply) Work-related activities Social media Creative School-related activities Leisure activities	11 (91.6) 9 (75.0) 1 (8.3) 3 (25.0) 10 (83.3)	6 (66.6) 6 (66.6) 0 (0.0) 6 (66.6) 5 (55.5)	9 (90.0) 9 (90.0) 1 (10.0) 4 (40.0) 9 (90.0)	9 (100.0) 6 (66.7) 2 (22.2) 6 (66.7) 6 (66.7)
Access Websites for Health-related Activities? Yes No	11 (91.7) 1 (8.3)	6 (66.7) 3 (33.3)	10 (100.0) 0 (0.0)	7 (77.8) 2 (22.2)
Confidence in using health-related websites/apps (1–10)* Mean (SD)	7.8 (1.8)	7.7(1.4)	7.7(2.9)	7.7(1.2)
How do you access the internet? Mostly on my phone Mostly on a computer/laptop/tablet About equally on a phone/computer/laptop/tablet Neither phone or computer/laptop/tablet	6 (50.0) 2 (16.7) 4 (33.3) 0 (0.0)	2 (22.2) 3 (33.3) 4 (44.4) 0 (0.0)	6 (60.0) 0 (0.0) 4 (40.0) 0 (0.0)	4 (44.4) 2 (22.2) 3 (33.3) 0 (0.0)
Role Youth Parent Nurse Physician	2 (16.7) 3 (25.0) 5 (41.6) 2 (16.7)	2 (22.2) 3 (33.3) 1 (11.1) 3 (33.3)	0 (0.0) 1 (10.0) 7 (70.0) 2 (20.0)	1 (11.1) 3 (33.3) 1 (11.1) 4 (44.4)

*Some participants chose not to answer this question

#### Asthma tool

**Fig. 1:**
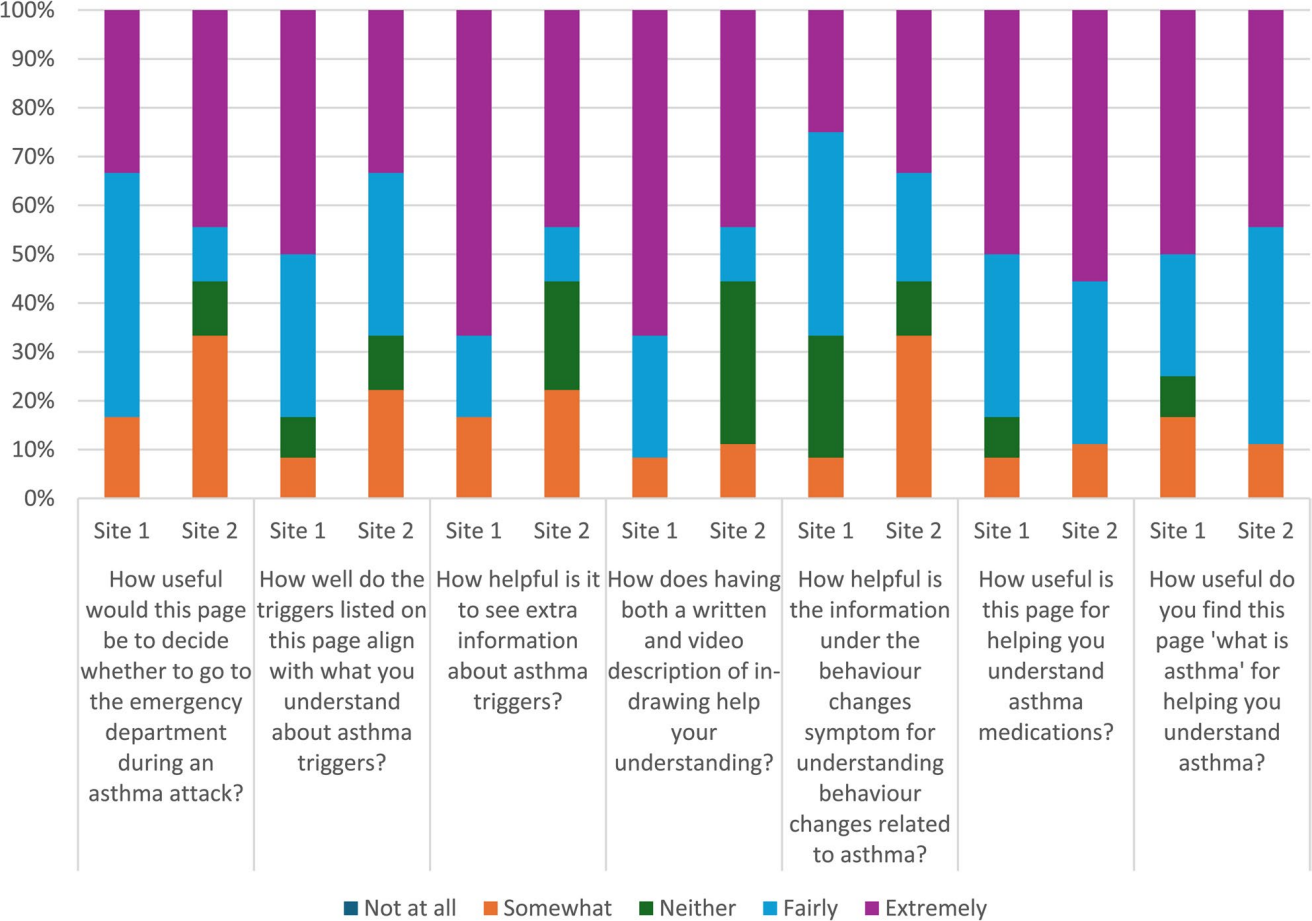
Usability Testing Round 2 results—asthma survey results

Asthma Usability Testing Round 2 results stratified by Site are presented in Fig. 1. Overall, no participants found any components of the tool unhelpful. Participants from Site 1 were more likely than Site 2 to find the tool helpful at informing when to visit the PED during an asthma attack, informing about asthma triggers, and using multiple modalities for describing asthma triggers (i.e., in-drawing). In contrast, Site 2 participants were more likely than Site 1 to feel neutrally about the additional asthma triggers information and found the information about behaviour changes for asthma symptoms somewhat helpful.

Qualitative data from Round 2 were categorized using the same three groups as Round 1: (i) overall satisfaction; (ii) decision supports and content; and (iii) suggestions for improvement. A brief narrative description for each of these categories is outlined below, with more detailed participant quotes found in Supplementary File 5.

- *Overall Satisfaction*. Participants generally liked the tool and described finding it helpful when deciding when to attend the PED during an asthma attack. Site 2 participants generally found the traffic light decision tree more useful than Site 1 participants.
- *Decision Supports and Content*. Participants were generally positive about the usefulness and helpfulness of the information found on the triggers page. Of the 21 asthma participants across both sites, 16 (76.2%) found it useful having written and visual descriptions of the in-drawing symptom.
- *Suggestions for Improvement*. Participants provided additional constructive suggestions to improve the new ‘medications’ and ‘what is asthma’ pages. Several participants highlighted the need for information about the aerochamber on the medication page and for better visuals on the asthma education page.

### Concussion tool

**Fig. 2:**
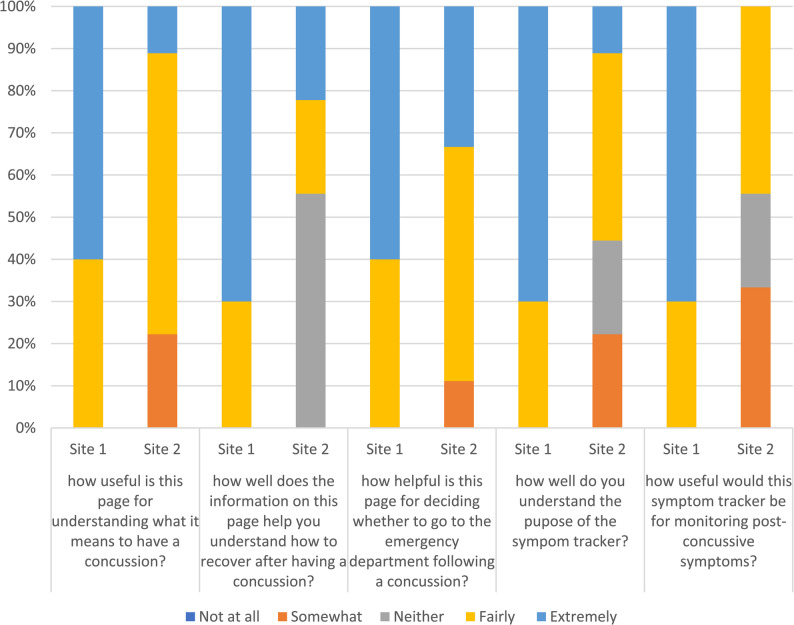
Usability Testing Round 2- concussion survey results

No concussion participants found any aspects of the tool unhelpful or not useful (Fig.  2). Generally, Site 1 respondents more often rated the concussion tool as extremely helpful and useful across all five survey items compared to Site 2 participants. Site 2 participants were more likely to select ‘somewhat useful/helpful’ for four of the five survey items and found the concussion recovery information page neither helpful nor unhelpful compared to Site 1.

Qualitative responses from concussion tool surveys were coded and analyzed using the same three categories as the asthma tool. A narrative description of each category is outlined below, with representative quotes outlined in Supplementary File 5.

- *Overall Satisfaction*. Most participants (n=17/19; 89.5%) rated the concussion information page as useful and appreciated the information was presented using lay language.
- *Decision Supports and Content*. Participants (n=14/19; 73.7%) rated the ‘concussion recovery page’ as helpful, with less favourable responses from Site 2 participants. Participants described that this page was simple and clear for describing concussion recovery. All participants but one (n=18/19; 94.7%) rated the tool feature that helps patients decide to go to the PED as helpful. However, qualitative responses provided some ideas for improvement, such as including more descriptors around certain red flag symptoms. There were mixed results from participants when asked about the symptom tracker component of the tool. About half of Site 2 participants (n=4/9;44.4%) reported a slight understanding the purpose of this feature, while all Site 1 participants reported a good understanding this aspect. Similarly, Site 2 participants did not find this feature as useful as Site 1 participants, with five participants rating it as somewhat or neither useful/not useful from Site 2, compared to all Site 1 participants finding it useful.
- *Suggestions for Improvement*. Clinicians from both sites provided comments which highlighted the need to make the tool accessible to different audiences, and available in different languages. Participants also suggested how the information could clear up any misinformation for parents around concussion, such as letting someone with a concussion fall asleep.

**Fig. 3:**
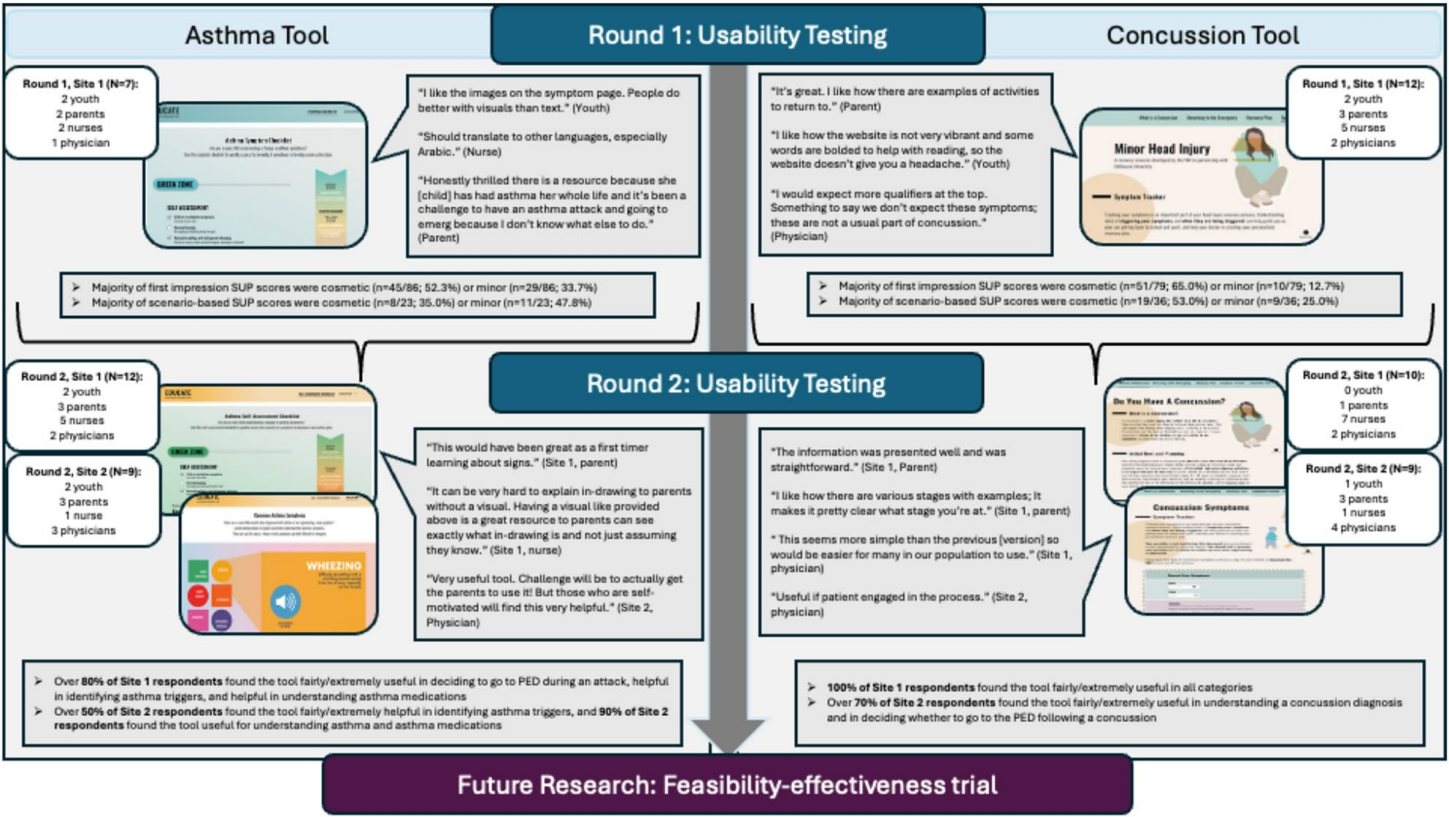
Summary of the results and progression of tool development

A summary of the results and progression of tool development across both rounds of usability testing, with exemplar quotes and quantitative input can be found in Fig. 3.

## Discussion

This study was a novel exploration of the usability of two electronic discharge communication tools for use in the PED for asthma and concussion presentations. Each tool was positively received by youth, parent, and clinician participants and were considered a useful addition to PED discharge care. This aligns with evidence that personalization, patient empowerment and self-management are essential to successful digital health tool uptake [35]. Therefore, including youth, parents, and PED clinicians in co-designing and testing of these tools likely contributed to the high level of user satisfaction. These refined tools are now well suited to be tested in a feasibility-effectiveness trial at PED sites across Canada.

The asthma tool was developed to help individuals decide whether to go to the PED during an asthma attack. Participants across both rounds of usability testing correctly described the purpose of this tool and generally thought the tool would be helpful to inform their decision making. Usability Testing Round 1 asthma participants suggested including more specific details about asthma symptoms, while Round 2 asthma participants requested more information and visuals for proper medication use. Poor education about asthma and improper medication use have been associated with uncontrolled asthma and frequent return PED visits [36]. Similarly, the sound clips and videos of symptoms were seen as particularly useful. This is unsurprising as multimedia formats can be more successful at improving comprehension at discharge compared to standard written formats [37], highlighting the importance of incorporating these suggestions in future iterations of the tool.

The concussion tool was co-designed to support post-concussion symptom management following PED discharge. This tool was well received in both rounds of usability testing, with similar feedback from each participant group in Round 1 and across both sites in Round 2. However, it is important to note that Round 2 included few youth and parent participants, particularly from Site 2. This low participation rate is likely due to the tight eligibility criteria for concussion participants, and will be important to address through future feasibility trials. Despite the limited engagement with youth and parents in Round 2, there was good representation and feedback from these groups for the initial version of the tool, and we exceeded the target goal for clinician participants. Clinicians suggested the concussion tool may be useful to clear up misinformation about concussion recovery. Assessments of online concussion content for youth revealed a high level of non-evidence based, anecdotal content [38], highlighting the potential value of including an educational component in our tool. Other research has shown an educational, text-messaging intervention for post-discharge concussion care was highly acceptable to parents [39], and may be a promising approach for enhancing the concussion tool.

Despite attempting to recruit a diverse sample of participants in each round of usability testing, most participants were Caucasian (*n* = 41/54; 76%) and spoke English as their first language. There was also a large proportion of clinician participants in Round 2 (*n* = 25/40; 62.5%) compared to Round 1 (*n* = 7/14; 50%). This is an important consideration as the tool is intended to be used mainly by parents and youth, with clinician input. Despite this discrepancy between rounds of testing, Round 1 required more in-depth feedback and tool changes, and was therefore well informed by youth and parent voices. Interestingly, Usability Testing Round 2, Site 2 participants were more diverse than Site 1 participants, and Site 2 participants were less likely to score the tools as useful or valuable compared to Site 1. It is unclear whether these variations in scores are due to differences in demographic characteristics of participants or other factors, such as the availability of other tools at each PED site. Clinician participants in each round clearly recognized the need to incorporate equity and diversity into PED discharge material by suggesting tools be offered in multiple languages and formats. However, future work is needed to enhance recruitment of diverse populations in testing health information interventions to ensure their unique needs are being identified and addressed.

A key strength of this study was its foundation in co-design methodology. Our original co-design team members were consulted and involved in each step of the intervention design and usability testing process, including adapting each tool after Usability Testing Round 1. A limitation of this study were the recruitment challenges. As Usability Testing Round 1 was primarily conducted during a period of strict COVID-19 public health measures in Canada, this may have limited the number of people seeking treatment at PEDs and constrained our potential participant pool. We also had narrow eligibility criteria due to the need to recruit youth and parents with specific PED experiences which likely further limited who could participate. However, the number of participants still allowed for identifying the key usability issues in each tool [29]. The overburdened health system, especially in the PED during the study period, made it difficult to recruit clinicians. These recruitment issues led to us adapting Usability Testing Round 2 to use remote, asynchronous testing which facilitated participation. Another limitation was that Round 2 testing of the concussion tool had only five (26.3%) youth/parent participants. Therefore, it is difficult to know whether feedback from this round is truly representative of youth and parent perspectives. As it can be challenging to actively engage youth in health research [40–42] using participatory research approaches to involve this demographic in future studies is needed to ensure their perspectives are included in health intervention design [43]. Finally, the survey used in Usability Testing Round 2 was briefly infiltrated by spam accounts. Due to frequent monitoring, the issue was resolved quickly, and the survey was closed. Data points were reviewed by the research staff, but it is possible some legitimate data may have been discarded in this process.

## Conclusion

This study provided novel insight into usability testing outcomes for two electronic discharge communication tools for asthma and concussion. By incorporating feedback from youth, parents, and clinicians from two Canadian PED sites, across two rounds of testing, we developed electronic discharge communication tools for asthma and concussion that are acceptable to different end-users. Next steps should focus on feasibility testing of these tools in multiple PED sites, with special attention on diversity and inclusion to ensure the tools meet a range of information needs at PED discharge.

## Supplementary Information

Supplementary Material 1.

Supplementary Material 2.

Supplementary Material 3.

Supplementary Material 4.

Supplementary Material 5.

## Data Availability

The datasets used and/or analyzed during the current study are available from the corresponding author on reasonable request.

## Abbreviations

ED: Emergency Department
EDUCATE: Emergency Department Discharge Communication Strategies
GRAMMS: Good Reporting of A Mixed Methods Study
PED: Pediatric Emergency Department
REDCap: Research Electronic Data Capture
SD: Standard Deviation
SUP: Severity of Usability Problems
