# Supplementary material for: Usability testing of two co-designed discharge communication tools for use in pediatric emergency departments: findings from the EDUCATE study
Source: BMC Pediatr. 2026 Apr 23;26:536. doi: 10.1186/s12887-026-06916-1 (PMC13244825; doi:10.1186/s12887-026-06916-1)
Supplement: Supplementary file 1 — Supplementary Material 1. [file 12887_2026_6916_MOESM1_ESM.docx]

**Supplementary Table- Additional Qualitative Feedback from Usability Testing Round 1- Asthma Tool**

| **Theme** | **Additional Quotes** |
| --- | --- |
| Overall Satisfaction | “*Honestly thrilled there is a resource because she [child] has had asthma her whole life and it’s been a challenge to have an asthma attack and going to emerg because I don’t know what else to do.”* (Parent)  “*Relevant tool, some changes suggested but overall, I like the concept”* (Parent)  “*I think it is a great tool and expressed my main thoughts throughout the study.”* (Youth)  “*Gathering that if two things are clicked that everything is good…I assume, although not totally clear if something isn’t correct in the green zone, I would go down to yellow zone? What’s not totally clear is why ‘this one’ is clicked while others are not…Still unsure why none of these are clicked in the ED zone, but they are checked in the yellow zone*.” (Parent) |
| Decision Support & Content | *“Traffic light flag should be bigger.”* (Youth)  *“I like the images on the symptom page. People do better with visuals than text. Images are effective – Bang on!”* (Youth)  *“Symptom page is helpful for parent education as they often don’t know what counts as a symptom.”* (Nurse)  *“Under triggers, can add interactive buttons with more details. For example instead of cigarettes can say ‘smoke’ and include cigarettes, wood stoves, etcetera.”* (Nurse)  *“Should add a separate education page on what is asthma and what medications are most common.”* (Physician)  *“Should add information about medication and how to properly give the inhaler under education tab.”* (Nurse)  *“Like the interactive feature of the symptoms page. Sound clips are really helpful.”* (Nurse)  *“Add details about if they use a medication like steroid that it might take time. Treatment needs to continue for a certain length of time.”* (Physician) |
| Suggestions for Improvement | *“We need to consider our lower intellect and low socioeconomic population in wording and understanding.”* (Nurse)  *“Should translate to other languages, especially Arabic.”* (Nurse)  *“Need age specific tools.”* (Physician)  *“The online tool may need to be different from paper tool. Make sure readability level is appropriate.”* (Physician) |

**Additional Qualitative Feedback from Usability Round 1- Concussion Tool**

| **Theme** | **Additional Quotes** |
| --- | --- |
| Overall Satisfaction | *“It’s great. I like how there are examples of activities to return to.”* (Parent)  *“Really like how it says it guides you back to school and sport.”* (Youth)  *“Parents with kids with head injury are always asking a lot of questions so it’s nice they have somewhere they can go for info. Sometimes they forget what they were told so they can read it on their own time.”* (Nurse)  *“I think it’s great because it’s so self-explanatory, they can have some autonomy. They don’t need me there to do it.”* (Nurse)  “I really. Really like the symptom tracker, especially with head injury if your memory is foggy, being able to do that in the moment is great.” (Nurse) |
| Decision Supports & Content | *“For me the font is too big but probably good for others with poor eyesight or concussion.”* (Nurse)  *“Not sure if I like that language ‘shaking of the head’. It is kind of shaking but maybe different language that could be used there. I know the medical terms but if a parent was reading this they might think it’s the head shaking visually rather than the brain shaking.”* (Nurse)  “I like how the website is not very vibrant and some words are bolded to help with reading, so the website doesn’t give you a headache.” (Youth)  “Unless given specific instructions to track head injury after leaving the ED, I would not open the symptom tracker tab. I like the symptom tracker but need more context as to what it is for and why I’m using it.” (Parent) |
| Suggestions for Improvement | *“It would help if there was some more detail about the symptoms like vomiting six times, not eating food.”* (Parent)  *“I would expect more qualifiers at the top. Something to say we don’t expect these symptoms, these are not a usual part of concussion.”* (Physician)  “I would want mnore description under ‘vomiting repeatedly’ to help decide to go back to ED. Like number of times or number of days.” (Youth) |
