# Supplementary figures and images for: Usability testing of two co-designed discharge communication tools for use in pediatric emergency departments: findings from the EDUCATE study
Source: BMC Pediatr. 2026 Apr 23;26:536. doi: 10.1186/s12887-026-06916-1 (PMC13244825; doi:10.1186/s12887-026-06916-1)

**Supplementary File 1.** Screenshots of each tool


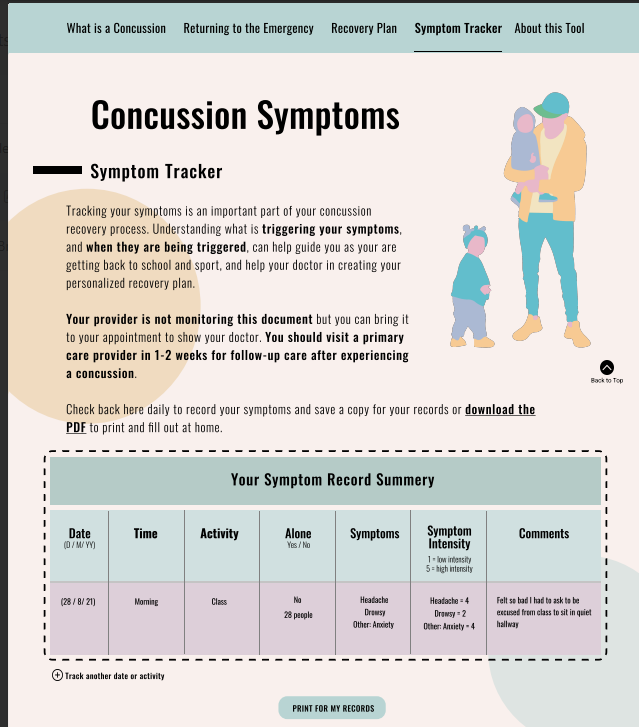


**
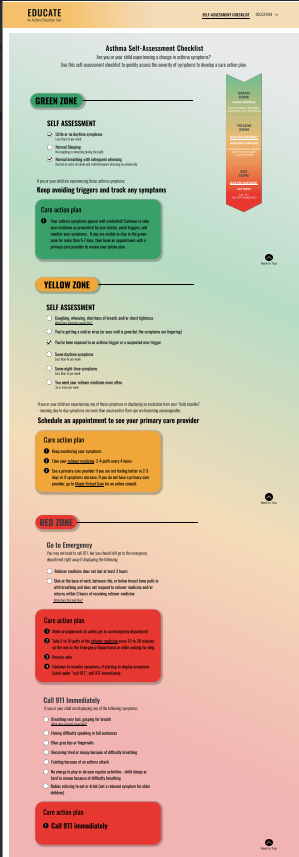
**

Supplement: Supplementary file 2 — Supplementary Material 2. [file 12887_2026_6916_MOESM2_ESM.docx]
