# Supplementary material for: Usability testing of two co-designed discharge communication tools for use in pediatric emergency departments: findings from the EDUCATE study
Source: BMC Pediatr. 2026 Apr 23;26:536. doi: 10.1186/s12887-026-06916-1 (PMC13244825; doi:10.1186/s12887-026-06916-1)
Supplement: Supplementary file 3 — Supplementary Material 3. [file 12887_2026_6916_MOESM3_ESM.docx]

**Supplementary File 3.** Usability Testing Round 1Post-test survey results

| **Question** | **Asthma (n=7)**  **N(%)** | **Concussion (n=7)**  **N(%)** |
| --- | --- | --- |
| Parents and Youth (n=7) | N=4 | N=3 |
| At first glance it attracted my attention  Strongly disagree  Disagree  Neither Agree/Disagree  Agree  Strongly Agree  N/A | 0 (0.0)  0 (0.0)  1 (25.0)  2 (50.0)  1 (25.0)  0 (0.0) | 0 (0.0)  0 (0.0)  2 (66.7)  0 (0.0)  1 (33.3)  0 (0.0) |
| It held my attention  Strongly disagree  Disagree  Neither Agree/Disagree  Agree  Strongly Agree  N/A | 0 (0.0)  1 (25.0)  0 (0.0)  1 (25.0)  2 (50.0)  0 (0.0) | 0 (0.0)  0 (0.0)  1 (33.3)  1 (33.3)  1 (33.3)  0 (0.0) |
| It is useful  Strongly disagree  Disagree  Neither Agree/Disagree  Agree  Strongly Agree  N/A | 0 (0.0)  0 (0.0)  0 (0.0)  3 (75.0)  1 (25.0)  0 (0.0) | 0 (0.0)  0 (0.0)  0 (0.0)  1 (33.3)  2 (66.7)  0 (0.0) |
| I like the illustrations  Strongly disagree  Disagree  Neither Agree/Disagree  Agree  Strongly Agree  N/A | 0 (0.0)  0 (0.0)  0 (0.0)  2 (50.0)  2 (50.0)  0 (0.0) | 0 (0.0)  1 (33.3)  1 (33.3)  1 (33.3)  0 (0.0)  0 (0.0) |
| I believe what it has to say  Strongly disagree  Disagree  Neither Agree/Disagree  Agree  Strongly Agree  N/A | 0 (0.0)  0 (0.0)  0 (0.0)  1 (25.0)  3 (75.0)  0 (0.0) | 0 (0.0)  0 (0.0)  0 (0.0)  1 (33.3)  2 (66.7)  0 (0.0) |
| I would recommend it to a friend or relative  Strongly disagree  Disagree  Neither Agree/Disagree  Agree  Strongly Agree  N/A | 0 (0.0)  0 (0.0)  1 (25.0)  0 (0.0)  3 (75.0)  0 (0.0) | 0 (0.0)  0 (0.0)  0 (0.0)  0 (0.0)  3 (100.0)  0 (0.0) |
| It is easy to understand  Strongly disagree  Disagree  Neither Agree/Disagree  Agree  Strongly Agree  N/A | 0 (0.0)  0 (0.0)  1 (25.0)  2 (50.0)  1 (25.0)  0 (0.0) | 0 (0.0)  0 (0.0)  1 (33.3)  0 (0.0)  2 (66.7)  0 (0.0) |
| What it says is important  Strongly disagree  Disagree  Neither Agree/Disagree  Agree  Strongly Agree  N/A | 0 (0.0)  0 (0.0)  0 (0.0)  0 (0.0)  4 (100.0)  0 (0.0) | 0 (0.0)  0 (0.0)  0 (0.0)  0 (0.0)  3 (100.0)  0 (0.0) |
| It reminds me of some things I need to think about  Strongly disagree  Disagree  Neither Agree/Disagree  Agree  Strongly Agree  N/A | 0 (0.0)  0 (0.0)  0 (0.0)  1 (25.0)  3 (75.0)  0 (0.0) | 0 (0.0)  0 (0.0)  0 (0.0)  2 (66.7)  1 (33.3)  0 (0.0) |
| It gives me some new things to think about  Strongly disagree  Disagree  Neither Agree/Disagree  Agree  Strongly Agree  N/A | 0 (0.0)  0 (0.0)  0 (0.0)  2 (50.0)  2 (50.0)  0 (0.0) | 0 (0.0)  2 (66.7)  1 (33.3)  0 (0.0)  0 (0.0)  0 (0.0) |
| It changes some of my thinking  Strongly disagree  Disagree  Neither Agree/Disagree  Agree  Strongly Agree  N/A | 0 (0.0)  2 (50.0)  0 (0.0)  1 (25.0)  1 (25.0)  0 (0.0) | 0 (0.0)  1 (33.3)  2 (66.7)  0 (0.0)  0 (0.0)  0 (0.0) |
| It could change how I do things  Strongly disagree  Disagree  Neither Agree/Disagree  Agree  Strongly Agree  N/A | 0 (0.0)  0 (0.0)  0 (0.0)  2 (50.0)  2 (50.0)  0 (0.0) | 0 (0.0)  0 (0.0)  1 (33.3)  1 (33.3)  1 (33.3)  0 (0.0) |
| Overall, I recommend that doctors use this material with patients  Strongly disagree  Disagree  Neither Agree/Disagree  Agree  Strongly Agree  N/A | 0 (0.0)  0 (0.0)  0 (0.0)  1 (25.0)  3 (75.0)  0 (0.0) | 0 (0.0)  0 (0.0)  0 (0.0)  0 (0.0)  3 (100.0)  0 (0.0) |
| Overall, I am the right person to get this material from my doctor  Strongly disagree  Disagree  Neither Agree/Disagree  Agree  Strongly Agree  N/A | 0 (0.0)  0 (0.0)  1 (25.0)  1 (25.0)  2 (50.0)  0 (0.0) | 0 (0.0)  0 (0.0)  0 (0.0)  1 (33.3)  2 (66.7)  0 (0.0) |
| Overall, the material accomplishes its main purpose  Strongly disagree  Disagree  Neither Agree/Disagree  Agree  Strongly Agree  N/A | 0 (0.0)  0 (0.0)  0 (0.0)  2 (50.0)  2 (50.0)  0 (0.0) | 0 (0.0)  0 (0.0)  0 (0.0)  2 (66.7)  1 (33.3)  0 (0.0) |
| It was helpful to see another youth do a think aloud example (youth only; n=2 & n=2)  Strongly disagree  Disagree  Neither Agree/Disagree  Agree  Strongly Agree  N/A | 0 (0.0)  0 (0.0)  1 (50.0)  0 (0.0)  1 (50.0)  0 (0.0) | 0 (0.0)  1 (33.3)  0 (0.0)  1 (0.0)  0 (0.0)  0 (0.0) |
| Clinicians (n=7) | N=3 | N=4 |
| The resource is designed to reinforce information  Strongly disagree  Disagree  Neither Agree/Disagree  Agree  Strongly Agree  N/A | 0 (0.0)  0 (0.0)  0 (0.0)  0 (0.0)  3 (100.0)  0 (0.0) | 0 (0.0)  0 (0.0)  1 (25.0)  0 (0.0)  3 (75.0)  0 (0.0) |
| The resource is designed to provide new information  Strongly disagree  Disagree  Neither Agree/Disagree  Agree  Strongly Agree  N/A | 0 (0.0)  0 (0.0)  0 (0.0)  2 (66.7)  1 (33.3)  0 (0.0) | 0 (0.0)  1 (25.0)  1 (25.0)  1 (25.0)  1 (25.0)  0 (0.0) |
| The resource is designed to stimulate behaviour change  Strongly disagree  Disagree  Neither Agree/Disagree  Agree  Strongly Agree  N/A | 0 (0.0)  0 (0.0)  1 (33.3)  1 (33.3)  1 (33.3)  0 (0.0) | 0 (0.0)  0 (0.0)  1 (25.0)  0 (0.0)  2 (50.0)  1 (25.0) |
| **Appearance** |  |  |
| At first glance it attracted my attention  Very low appeal  Low appeal  Neither low/high appeal  High appeal  Very high appeal  N/A | 0 (0.0)  1 (33.3)  1 (33.3)  1 (33.3)  0 (0.0)  0 (0.0) | 0 (0.0)  1 (25.0)  0 (0.0)  1 (25.0)  2 (50.0)  0 (0.0) |
| It held my attention  Very low appeal  Low appeal  Neither low/high appeal  High appeal  Very high appeal  N/A | 0 (0.0)  2 (66.67)  0 (0.0)  1 (25.0)  0 (0.0)  0 (0.0) | 0 (0.0)  0 (0.0)  0 (0.0)  2 (50.0)  2 (50.0)  0 (0.0) |
| Overall appearance  Very low appeal  Low appeal  Neither low/high appeal  High appeal  Very high appeal  N/A | 0 (0.0)  0 (0.0)  1 (33.3)  1 (33.3)  1 (33.3)  0 (0.0) | 0 (0.0)  0 (0.0)  1 (25.0)  1 (25.0)  2 (50.0)  0 (0.0) |
| Quality of illustration  Very low appeal  Low appeal  Neither low/high appeal  High appeal  Very high appeal  N/A | 0 (0.0)  0 (0.0)  0 (0.0)  2 (66.67)  1 (33.3)  0 (0.0) | 0 (0.0)  1 (25.0)  0 (0.0)  3 (75.0)  0 (0.0)  0 (0.0) |
| Use of colour  Very low appeal  Low appeal  Neither low/high appeal  High appeal  Very high appeal  N/A | 1 (33.3)  0 (0.0)  1 (33.3)  1 (33.3)  0 (0.0)  0 (0.0) | 1 (25.0)  0 (0.0)  0 (0.0)  0 (0.0)  3 (75.0)  0 (0.0) |
| Typeface (large enough; attractive)  Very low appeal  Low appeal  Neither low/high appeal  High appeal  Very high appeal  N/A | 0 (0.0)  0 (0.0)  1 (33.3)  1 (33.3)  1 (33.3)  0 (0.0) | 0 (0.0)  0 (0.0)  0 (0.0)  0 (0.0)  4 (100.0)  0 (0.0) |
| Highlighting of major concepts  Very low appeal  Low appeal  Neither low/high appeal  High appeal  Very high appeal  N/A | 0 (0.0)  0 (0.0)  0 (0.0)  3 (100.0)  0 (0.0)  0 (0.0) | 0 (0.0)  0 (0.0)  0 (0.0)  0 (0.0)  4 (100.0)  0 (0.0) |
| **Please rate the content of the material.** |  |  |
| Up-to-date  Very poor  Poor  Neither poor/good  Good  Very good  N/A | 0 (0.0)  0 (0.0)  1 (33.3)  1 (33.3)  1 (33.3)  0 (0.0) | 0 (0.0)  0 (0.0)  0 (0.0)  1 (25.0)  3 (75.0)  0 (0.0) |
| Scientifically accurate  Very poor  Poor  Neither poor/good  Good  Very good  N/A | 0 (0.0)  0 (0.0)  0 (0.0)  2 (66.7)  1 (33.3)  0 (0.0) | 0 (0.0)  0 (0.0)  1 (25.0)  1 (25.0)  2 (50.0)  0 (0.0) |
| Adequate scope for objectives  Very poor  Poor  Neither poor/good  Good  Very good  N/A | 0 (0.0)  0 (0.0)  1 (33.3  1 (33.3)  1 (33.3)  0 (0.0) | 0 (0.0)  0 (0.0)  0 (0.0)  2 (50.0)  2 (50.0)  0 (0.0) |
| Overall organization  Very poor  Poor  Neither poor/good  Good  Very good  N/A | 0 (0.0)  0 (0.0)  0 (0.0)  2 (66.7)  1 (33.3)  0 (0.0) | 0 (0.0)  0 (0.0)  0 (0.0)  1 (25.0)  3 (75.0)  0 (0.0) |
| Logical flow of ideas  Very poor  Poor  Neither poor/good  Good  Very good  N/A | 0 (0.0)  0 (0.0)  1 (33.3)  1 (33.3)  1 (33.3)  0 (0.0) | 0 (0.0)  0 (0.0)  0 (0.0)  3 (75.0)  1 (25.0)  0 (0.0) |
| Needed background given to enable understanding  Very poor  Poor  Neither poor/good  Good  Very good  N/A | 0 (0.0)  0 (0.0)  0 (0.0)  3 (100.0)  0 (0.0)  0 (0.0) | 0 (0.0)  0 (0.0)  2 (50.0)  0 (0.0)  2 (50.0)  0 (0.0) |
| Summaries given when needed  Very poor  Poor  Neither poor/good  Good  Very good  N/A | 0 (0.0)  0 (0.0)  2 (66.7)  0 (0.0)  1 (33.3)  0 (0.0) | 0 (0.0)  0 (0.0)  0 (0.0)  2 (50.0)  1 (25.0)  1 (25.0) |
| The management of fear content  Very poor  Poor  Neither poor/good  Good  Very good  N/A | 0 (0.0)  1 (33.3)  1 (33.3)  0 (0.0)  1 (33.3)  0 (0.0) | 0 (0.0)  2 (50.0)  0 (0.0)  0 (0.0)  2 (50.0)  0 (0.0) |
| Fair presentation given (e.g., avoids sexism, ethnic bias, ageism)  Very poor  Poor  Neither poor/good  Good  Very good  N/A | 0 (0.0)  1 (33.3)  1 (33.3)  1 (33.3)  0 (0.0)  0 (0.0) | 0 (0.0)  0 (0.0)  0 (0.0)  1 (25.0)  2 (50.0)  1 (25.0) |
| If a fair presentation is not given: Does the bias interfere with the intent of the item?  Yes  No  N/A | 0 (0.0)  2 (66.7)  1 (33.3) | 0 (0.0)  4 (100.0)  0 (0.0) |
| **Usefulness** |  |  |
| It is useful for its intended audience  Strongly disagree  Disagree  Neither Agree/Disagree  Agree  Strongly Agree  N/A | 0 (0.0)  0 (0.0)  1 (33.3)  0 (0.0)  2 (66.7)  0 (0.0) | 0 (0.0)  0 (0.0)  0 (0.0)  1 (25.0)  3 (75.0)  0 (0.0) |
| It is believable  Strongly disagree  Disagree  Neither Agree/Disagree  Agree  Strongly Agree  N/A | 0 (0.0)  0 (0.0)  0 (0.0)  1 (33.3)  2 (66.7)  0 (0.0) | 0 (0.0)  0 (0.0)  0 (0.0)  1 (25.0)  3 (75.0)  0 (0.0) |
| The material is understandable  Strongly disagree  Disagree  Neither Agree/Disagree  Agree  Strongly Agree  N/A | 0 (0.0)  0 (0.0)  1 (33.3)  2 (66.7)  0 (0.0)  0 (0.0) | 0 (0.0)  0 (0.0)  0 (0.0)  1 (25.0)  3 (75.0)  0 (0.0) |
| Requires little or no explanation  Strongly disagree  Disagree  Neither Agree/Disagree  Agree  Strongly Agree  N/A | 0 (0.0)  2 (66.7)  0 (0.0)  1 (33.3)  0 (0.0)  0 (0.0) | 0 (0.0)  1 (25.0)  1 (25.0)  0 (0.0)  2 (50.0)  0 (0.0) |
| **Overall** |  |  |
| Overall, I would recommend that physicians use this material with patients  Strongly disagree  Disagree  Neither Agree/Disagree  Agree  Strongly Agree  N/A | 0 (0.0)  0 (0.0)  1 (33.3)  1 (33.3)  1 (33.3)  0 (0.0) | 0 (0.0)  0 (0.0)  0 (0.0)  1 (25.0)  3 (75.0)  0 (0.0) |
| Overall, this material meets its objectives  Strongly disagree  Disagree  Neither Agree/Disagree  Agree  Strongly Agree  N/A | 0 (0.0)  0 (0.0)  0 (0.0)  1 (33.3)  1 (33.3)  1 (33.3) | 0 (0.0)  0 (0.0)  0 (0.0)  2 (50.0)  2 (50.0)  0 (0.0) |
| **Placement** |  |  |
| Static version should be placed in the emergency department  Strongly disagree  Disagree  Neither Agree/Disagree  Agree  Strongly Agree  N/A | 0 (0.0)  0 (0.0)  1 (33.3)  0 (0.0)  2 (66.7)  0 (0.0) | 0 (0.0)  0 (0.0)  0 (0.0)  0 (0.0)  4 (100.0)  0 (0.0) |
| Should be incorporated into an existing resource  Strongly disagree  Disagree  Neither Agree/Disagree  Agree  Strongly Agree  N/A | 0 (0.0)  0 (0.0)  0 (0.0)  0 (0.0)  2 (66.7)  1 (33.3) | 0 (0.0)  0 (0.0)  2 (50.0)  0 (0.0)  2 (50.0)  0 (0.0) |
| Should be provided at the beginning of the visit (i.e., during triage)  Strongly disagree  Disagree  Neither Agree/Disagree  Agree  Strongly Agree  N/A | 0 (0.0)  1 (33.3)  0 (0.0)  1 (33.3)  0 (0.0)  1 (33.3) | 1 (25.0)  2 (50.0)  1 (25.0)  0 (0.0)  0 (0.0)  0 (0.0) |
| Should be provided at the end of the visit (i.e., during discharge)  Strongly disagree  Disagree  Neither Agree/Disagree  Agree  Strongly Agree  N/A | 0 (0.0)  0 (0.0)  0 (0.0)  1 (33.3)  2 (66.7)  0 (0.0) | 0 (0.0)  0 (0.0)  0 (0.0)  1 (25.0)  3 (75.0)  0 (0.0) |
| Should be provided upon patients’ first presentation of [asthma/concussion]  Strongly disagree  Disagree  Neither Agree/Disagree  Agree  Strongly Agree  N/A | 0 (0.0)  0 (0.0)  0 (0.0)  2 (66.7)  1 (33.3)  0 (0.0) | 0 (0.0)  0 (0.0)  0 (0.0)  0 (0.0)  4 (100.0)  0 (0.0) |
| Should be provided upon patients’ follow-up visits  Strongly disagree  Disagree  Neither Agree/Disagree  Agree  Strongly Agree  N/A | 0 (0.0)  0 (0.0)  0 (0.0)  1 (33.3)  2 (66.7)  0 (0.0) | 0 (0.0)  0 (0.0)  0 (0.0)  1 (25.0)  2 (50.0)  1 (25.0) |
