# Supplementary material for: Usability testing of two co-designed discharge communication tools for use in pediatric emergency departments: findings from the EDUCATE study
Source: BMC Pediatr. 2026 Apr 23;26:536. doi: 10.1186/s12887-026-06916-1 (PMC13244825; doi:10.1186/s12887-026-06916-1)
Supplement: Supplementary file 4 — Supplementary Material 4. [file 12887_2026_6916_MOESM4_ESM.docx]

**Supplementary Table- Additional Qualitative Feedback from Usability Testing Round 2- Asthma Tool**

| **Theme** | **Additional Quotes** |
| --- | --- |
| Overall Satisfaction | *“Very useful tool. Challenge will be to actually get the parents to use it! But those who are self-motivated will find this very helpful.”* (Site 2, physician)  *“It is very similar to the discharge tool parents receive their child’s asthma prescription on.”* (Site 1, nurse)  “This would have been excellent as a first-time asthma parent, however, these are all signs and triggers that we have been explained on subsequent trips to the hospital.” (Site 1, parent) |
| Decision Support & Content | *“My daughter is fortunate that her asthma is mostly exacerbated by viruses/illnesses so we haven’t experienced the other causes.”* (Site 1, parent)  *“I remember the staff looking for that in my child when we went to the ED but didn’t know it was called in-drawing, so this cleared the definition right up.”* (Site 1, parent)  *“This would have been great as a first timer learning about signs.”* (Site 1, parent)  *“I think the visual video helps people be able to see it first-hand and not have to question what they are seeing when it happens for the first time at home with their child.”* (Site 1, nurse)  *“I think it is very helpful for parents to know that if their child has a viral illness with cough/congestion, they should be using Ventolin to prevent a severe asthma exacerbation requiring an ED visit.”* (Site 2, physician)  *“It can be very hard to explain in-drawing to parents without a visual. Having a visual like provided above is a great resource so parents can see exactly what in-drawing is and not just assuming they know.”* (Site 1, nurse |
| Suggestions for Improvement | “*Would suggest including info about air chamber, which most under 6-year-olds use.”* (Site 1, parent)  *“Image doesn’t seem relevant [on asthma education page.”* (Site 1, physician)  *“Maybe a picture showing a cross-section of a normal airway compared to an inflamed, mucousy, broncho-constricted airway would be helpful.”* (Site 2, physician)  *“I think it is missing the aerochamber component to puffers, which is essential if wanting the puffers to work effectively. A lot of parents/patients don’t always realize how essential the aerochamber is in letting the medication from the puffer get deep into the lungs and actually treat their symptoms.”* (Site 1, nurse) |

**Additional Qualitative Feedback from Usability Testing Round 2- Concussion Tool**

| **Theme** | **Additional Quotes** |
| --- | --- |
| Overall Satisfaction | *“Direct and to the point. Not too wordy.”* (Site 2, physician)  *“The information was presented well and was straightforward.”* (Site 1, parent)  *“I think this page appropriately uses simple language to explain the basics of what causes a concussion.”* (Site 2, physician) |
| Decision Supports & Content | *“Very similar to discharge advice I got from the adult ED regarding a concussion. I like the visual of this chart.”* (Site 1, nurse)  *“I like how there are various stages with examples; It makes it pretty clear what stage you’re at.”* (Site 1, parent)  *“I think the examples listed for each stage of recovery are especially important for patients to understand what they can and cannot expect to be doing activity-wise as they recover.”* (Site 2, physician)  *“This seems more simple than the previous so would be easier for many in our population to use.”* (Site 1, physician)  *“My only hesitation is ‘headache becoming severe’; not sure if this should be explained more. I find parents bring children back if their headache is not fully resolved but is also not becoming worse. Maybe adding something a bit more descriptive would help.”* (Site 1, nurse)  *“I guess the biggest concern would be if it was the patient themselves filling it in the case of a teenager as screen time might aggravate things.”* (Site 1, nurse) |
| Suggestions for Improvement | “*Love it!”* (Site 1, nurse)  *“The ‘drowsy’ and ‘think you are having a seizure’ don’t seem helpful.”* (Site 1, physician)  *“Information is clearly outlined.”* (Site 1, parent)  *“Useful if patient engaged in the process.”* (Site 2, physician)  *“Some guidance on how often. For example, children with underlying anxiety who then use this tool daily for weeks may exacerbate underlying symptoms.”* (Site 2, nurse)  *“Pamphlets as well and in multiple languages or have the option to choose the language from a drop-down menu and print.”* (Site 1, nurse)  *“It might be helpful to reinforce in the first introduction section that there is no need for head imaging if a diagnosis of concussion is made. I have many parents asking for head imaging out of concern regarding the symptoms of concussion.”* (Site 2, physician)  *“I have found that parents still think they have to wake their children up frequently if they have a concussion. It is my understanding this is only a myth, if that is the case, a section addressing this or highlighting the importance of uninterrupted rest and sleep during a concussion may be useful.”* (Site 1, nurse)  *“Multiple languages, like English, French, and Arabic to start.”* (Site 2, physician)  *“More information about diagnosis about concussion, section of myths about concussion. For example, most people still believe you can’t let a child sleep with concussion.”* (Site 1, nurse) |
