# Supplementary material for: Usability testing of two co-designed discharge communication tools for use in pediatric emergency departments: findings from the EDUCATE study
Source: BMC Pediatr. 2026 Apr 23;26:536. doi: 10.1186/s12887-026-06916-1 (PMC13244825; doi:10.1186/s12887-026-06916-1)
Supplement: Supplementary file 5 — Supplementary Material 5. [file 12887_2026_6916_MOESM5_ESM.docx]

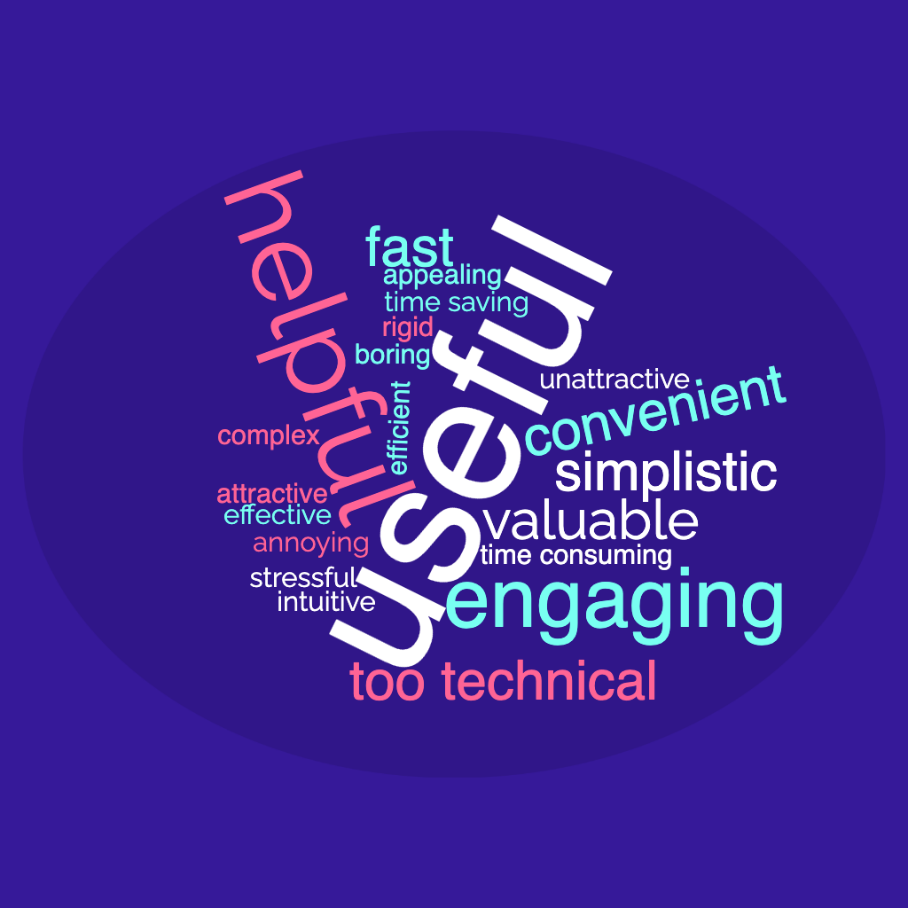


**Supplementary Figure 2.** Word cloud for the asthma tool from round 1 usability testing


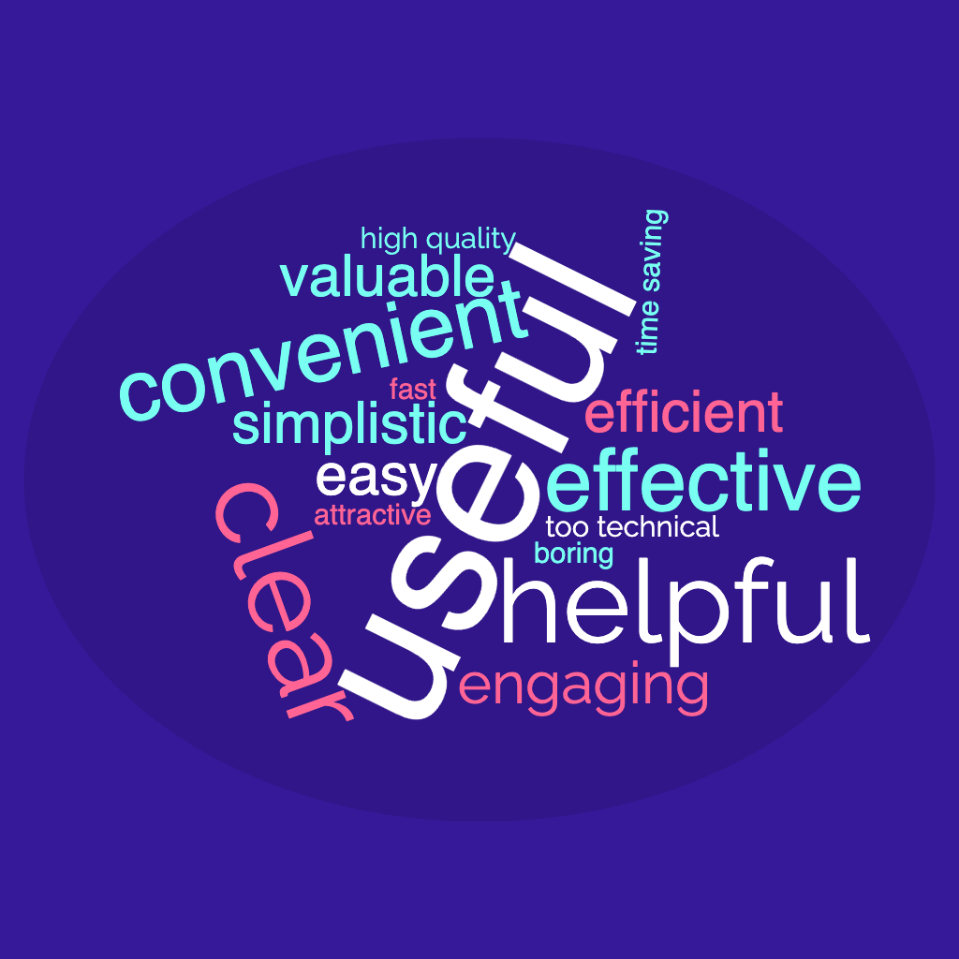


**Supplementary Figure 3.** Word cloud for the concussion tool from round 1 usability testing
